# High efficacy of PD-1 inhibitor after initial failure of PD-L1 inhibitor in Relapsed/Refractory classical Hodgkin Lymphoma

**DOI:** 10.1186/s12885-021-09028-4

**Published:** 2022-01-03

**Authors:** Xi Chen, Haiying Kong, Linxiang Luo, Shuiyun Han, Tao Lei, Haifeng Yu, Na Guo, Cong Li, Shuailing Peng, Xiaowu Dong, Haiyan Yang, Meijuan Wu

**Affiliations:** 1 Department of Lymphoma, Cancer Hospital of the University of Chinese Academy of Sciences (Zhejiang Cancer Hospital), Hangzhou, China; 2 Institute of Cancer and Basic Medicine (IBMC), Chinese Academy of Sciences, Hangzhou, China; 3 Department of Pharmacy, Zhejiang Medical and Health Group Hangzhou Hospital (Hangzhou Hanggang Hospital), Hangzhou, China; 4 Department of Hematology, Zhejiang Quhua Hospital, Quhua, China; 5 Department of Pathology, Cancer Hospital of the University of Chinese Academy of Sciences (Zhejiang Cancer Hospital), No. 1 Banshan East Road, Hangzhou, China; 6 Hangzhou Institute of Innovative Medicine, College of Pharmaceutical Sciences, Zhejiang University, Hangzhou, China

**Keywords:** Hodgkin lymphoma, PD-1 inhibitor, PD-L1 inhibitor, PD-L2, Molecular phenotype, Tumor microenvironment

## Abstract

**Purpose:**

We sought to understand the clinical course and molecular phenotype of patients who showed disease progression after programmed cell death ligand 1 (PD-L1) inhibitor treatment but subsequently responded to PD-1 inhibitor treatment. We also explored the response to PD-1-axis targeted therapy of classical Hodgkin lymphoma (cHL) according to genetically driven PD-L1 and programmed cell death ligand 2 (PD-L2) expression.

**Methods:**

Five patients in a phase II clinical trial of CS1001 (PD-L1 inhibitor) for relapsed or refractory (R/R) cHL were retrospectively reviewed. Formalin-fixed, paraffin-embedded whole tissues from the five patients were evaluated for 9p24.1 genetic alterations based on FISH and the expression of PD-L1, PD-L2, PD-1, major histocompatibility complex (MHC) class I–II, and the tumor microenvironment factorsCD163 and FOXP3 in the microenvironmental niche, as revealed by multiplex immunofluorescence.

**Results:**

All five patients showed primary refractory disease during first-line treatment. Four patients received PD-1 inhibitor after dropping out of the clinical trial, and all demonstrated at least a partial response. The progression-free survival ranged from 7 to 28 months (median = 18 months), and 9p24.1 amplification was observed in all five patients at the PD-L1/PD-L2 locus. PD-L1 and PD-L2 were colocalized on Hodgkin Reed-Sternberg (HRS) cells in four of the five (80%) patients. There was differential expression of PD-L1 and PD-L2 in cells in the tumor microenvironment in cHL, especially in HRS cells, background cells and tumor-associated macrophages.

**Conclusions:**

PD-L1 monotherapy may not be sufficient to block the PD-1 pathway; PD-L2 was expressed in HRS and background cells in cHL. The immunologic function of the PD-L2 pathway in anti-tumor activity may be underestimated in R/R cHL. Further study is needed to elucidate the anti-tumor mechanism of PD-1 inhibitor and PD-L1 inhibitor treatment.

**Supplementary Information:**

The online version contains supplementary material available at 10.1186/s12885-021-09028-4.

## Background

Hodgkin lymphoma is a relatively rare malignant disease that tends to have excellent outcomes. Doxorubicin, bleomycin, vinblastine and dacarbazine (ABVD) with or without radiotherapy is the most widely accepted first-line therapy for patients with classical Hodgkin lymphoma (cHL). Nonetheless, about 25% of patients relapse or experience a refractory event [1]. Second-line treatment followed by autologous stem cell transplantation (ASCT) is the standard approach for R/R cHL, but may not be appropriate for elderly and unfit patients. In addition, some patients may experience recurrence even after ASCT; new agents are needed to resolve this problem.

Tissues samples from cHL patients show sparse tumor cells (Hodgkin Reed-Sternberg [HRS] cells) in an inactive inflammatory/immune milieu. This observation led to the suggestion that it may be possible to reverse cellular immunosuppression in the tumor microenvironment (TME) to kill tumor cells. This led to research on immune therapies targeting the PD-1 axis.

The overexpression of programmed cell death ligand 1 (PD-L1) and programmed cell death ligand 2 (PD-L2) in HRS cells due to alterations in chromosome 9p24.1, PD-L1 (CD274) and PD-L2 (PDCD1LG2) induces ligands to bind PD-1 (CD279) on the surface of T cells to diminish their immune function [2]. Tumor-associated macrophages (TAMs) contribute the majority of PD-L1 in the TME and colocalize with PD-L1+ HRS cells, which are in extensive contact with PD-1+ T cells in the microenvironmental niche [3]. A meta-analysis showed that the efficacy of PD-1 or PD-L1 blockade differed significantly between patients who were PD-L1 positive and those who were not [4]. The blockade of the PD-1/PD-L1 immune checkpoint could be a treatment target for cHL.

Roemer et al. [2] evaluated PD-L1 and PD-L2 alterations using fluorescent in situ hybridization (FISH) and found that progression-free survival (PFS) was significantly shorter for patients exhibiting 9p24.1 amplification in cHL specimens. They also concluded that genetically driven PD-L1 expression of HRS cells are potential predictors of a favorable outcome in patients with R/R cHL [5]. A high proportion of PD-L1+ leukocytes [6] was also associated with inferior outcomes, indicating that PD-L1 can serve as a prognostic biomarker.

According to published papers, PD-L1 inhibitor did not bring out such good remission rate as PD-1 inhibitor did in patients with R/R cHL [7–9]. This suggests that molecular interactions with PD-1 and PD-L2 may also play an important role in cHL. Besides, other biomarkers such as the TME factors CD163 and FOXP3+ regulatory T cells (FOXP3+ Tregs) showed conflicting association with the outcome [10–13].

In the present work, we explored the molecular mechanism underlying treatment failure in cHL patients who received a PD-L1 inhibitor. In addition, we investigated the influence of expression patterns of PD-L1 and PD-L2 on PD-1 axis-targeted therapy. We also detected the expression of PD-1 and MHC class I–II in HRS cells, as well as the tumor microenvironment factors CD163 and FOXP3 in background cells.

## Methods

### Study population

We retrospectively reviewed the data of five patients from a phase II clinical trial of CS1001 (PD-L1 inhibitor) for R/R cHL (clincialtrials.gov identifier: NCT03505996, first registration date: 23/04/2018). They all showed primary refractory disease during first-line treatment, which was defined as end-of-treatment positron emission tomography-computed tomography (PET-CT) scan positive. The clinical and therapeutic data of these patients were collected from our clinical center. ABVD was given as first-line chemotherapy. All of the patients had been given several lines of chemotherapy, including BEACOPP, GPD, and ICE regimens with or without radiation, before CS1001 immunotherapy.

### Primary tumor specimens

Primary tumor specimens included five cHL specimens and one of normal tissue. Formalin-fixed, paraffin-embedded whole tissues from tumors were obtained from the archives of Zhejiang Cancer Hospital following institutional review board approval. Hematoxylin and eosin-stained tissue sections, as well as the original diagnostic reports were reviewed by a professional hematopathologist. Follow-up data were available up to April 2021.

### Fluorescence In-situ hybridization

First, 9p24.1 genetic alterations were evaluated by FISH assay; probes were purchased from Guangzhou Anbiping Pharmaceutical Technology Co., Ltd. (China) for PD-L1 (GPS PD-L1 CSP9, F.01256-01) and PD-L2 (GPS PD-L2 CSP9, F.01243). Copy number alterations were defined based on the target:control signal ratio according to the literature [5]. Fifty HRS cells per tumor tissue were analyzed. Nuclei with a target:control signal ratio of ≥ 3:1 were defined as amplified, and those with a signal ratio of > 1:1 but < 3:1 were considered as copy gain of these loci. Nuclei with a target:control signal ratio of 1:1 but more than two copies per probe were defined as polysomic for 9p24.1. For each patient, the magnitude and percentage of 9p24.1 amplification, copy gain, polysomy, and normal copy numbers (disomy) were recorded. Patients were classified based on the degree of 9p24.1 genetic alteration; those with 9p24.1 copy gain lacked amplification, and those with 9p polysomy lacked both 9p24.1 copy gain and amplification.

### Multiplex immunofluorescence

Multiplex immunofluorescence (mIF) was performed by staining 4-um-thick FFPE whole tissue sections using published protocols. The slides were scanned by a Pannoramic MIDI scanner (3DHISTECH, Hungary). The antibodies used were as follows: monoclonal mouse antibody against MHC class II (Abcam, UK); rabbit antibody against MHC class I (Invitrogen, USA); monoclonal mouse antibody against CD30 (Abcam); monoclonal mouse antibody against Foxp3 (Abcam); polyclonal rabbit antibody against PD-L1(Proteintech, USA); polyclonal rabbit antibody against PD-L2 (Abcam); monoclonal mouse antibody against CD68 (Servicebio, China); monoclonal mouse antibody against CD163 (Servicebio); and monoclonal mouse antibody against PD-1 (Servicebio). The percentages of cells staining for PD-1, PD-L1, PD-L2, CD68, MHC class I, MHC class II, FOXP3, and CD163 were denoted as follows: -, 0%; weak+, 1–5%; +, 6–10%; ++, 11–30%; +++, 31–60%; and ++++, > 60%.

### Follow-up

Overall survival was calculated from the time of diagnosis to death from any cause or the date of the last follow-up. Follow-up data were available up to April 2021.

## Results

### Patient characteristics

**Fig. 1:**
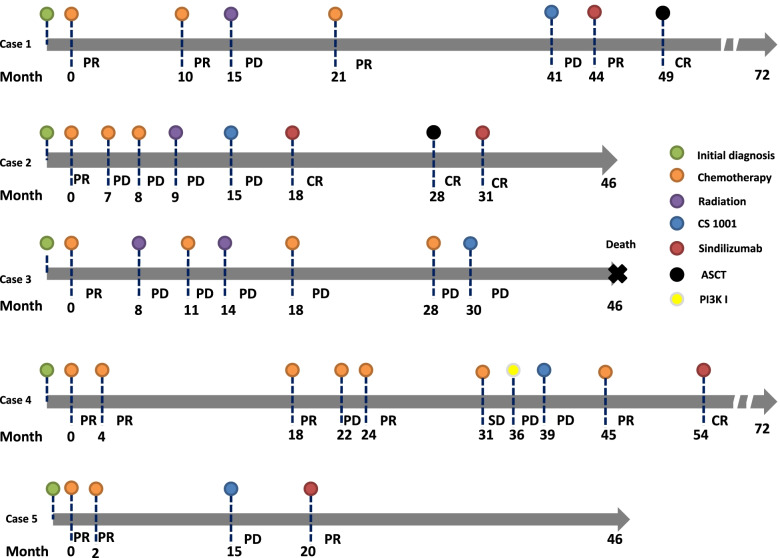
Treatment courses of the five patients

The characteristics and clinical course of the five patients are summarized in Table 1. All of the patients showed disease progression after several cycles of a PD-L1 inhibitor in a short time. Four patients were given an PD-1 inhibitor after they dropped out of the clinical trial, and all experienced dramatic and persistent responses (at least a PR). The PFS of anti-PD-1 therapy ranged from 18 to 28 months (median = 27 months) (Fig. 1). Patients 1 and 2 were assessed using PET-CT, which revealed metabolic remission (Supplementary Fig. 1 A and 1B) during treatment with anti-PD-1 therapy. Only patient 3 refused anti-tumor therapy and died 1 year after anti-PD-L1 therapy (Fig. 1).

**Table 1:** Clinical characteristics, therapeutic data and outcomes of the five patients

Patient	Gender	Age	Histopathology	Ann Arbor Stage	Primary refractory^a^	Lines of chemo before IT	PD-L1 inhibitor			PD-1 inhibitor			OS	Status
							Cycle	Response	Duration	Cycle	Response^b^	Duration		
1	M	24y	NSHL	IVA	Yes	3 + Rad	8	PD	3 m	13	CR	28 m	72 m	Alive
2	F	36y	NSHL	IVB	Yes	3 + Rad	4	PD	3 m	12	CR	28 m	46 m	Alive
3	F	54y	NSHL	IIA	Yes	4 + Rad	4	PD	3 m	-	-	-	46 m	Dead
4	F	43y	MCHL	IIA	Yes	7	6	PD	4 m	6	CR	18 m	72 m	Alive
5	M	33y	NSHL	IVA	Yes	2	8	PD	5 m	11	PR	26 m	46 m	Alive

Abbreviation: OS = overall survival; F = female; M = male; NSHL = nodular sclerosing Hodgkin lymphoma; MCHL = mixed cellularity Hodgkin lymphoma; IT = immunotherapy; Rad = radiation; CR = complete response; PR = partial response; PD = progressive disease

^a^Primary refractory was defined as end-of-treatment positron emission tomography-computed tomography (PET-CT) scan positive

^b^Patient 1, 2 and 4 were in CR, patient 5 was in PR at the last assessment

### Spectrum of 9p24.1 alterations in cHL

**Fig. 2:**
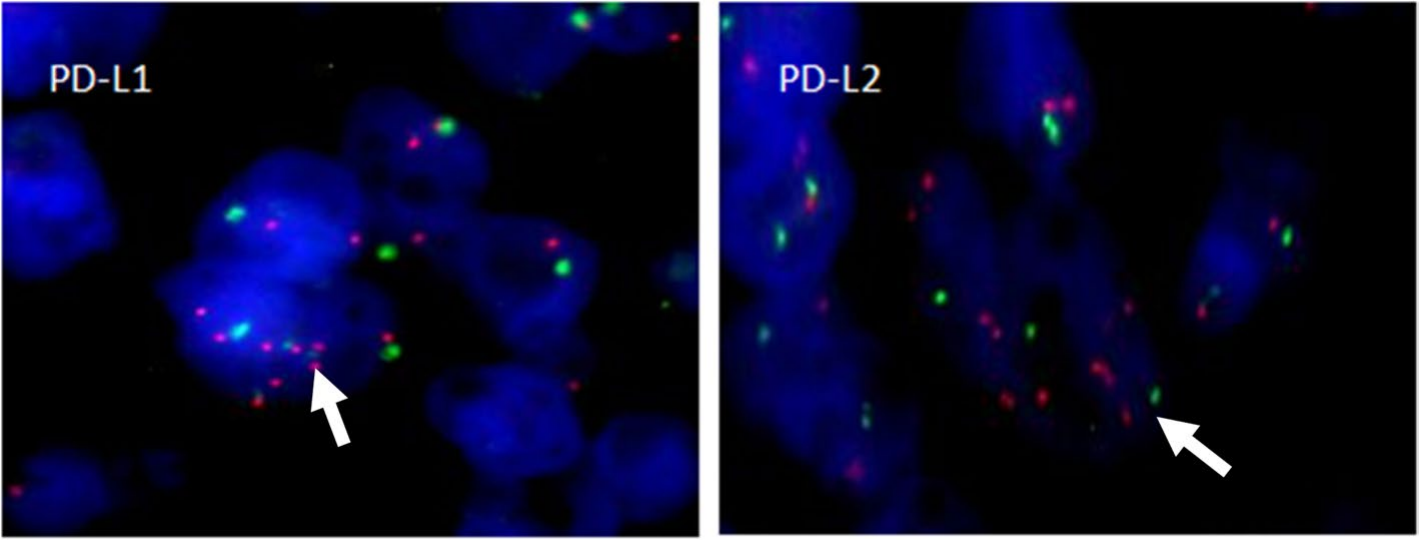
Alterations of the PD-L1/PD-L2 locus were investigated by FISH using a PD-L1/PD-L2 probe (labeled with Spectrum Red) and a CEP 9 probe (labeled with Spectrum Green). Representative FISH images revealed amplification of the PD-L1/PD-L2 locus in Patient 1, indicated by a red signal (white arrow)

We assessed the spectrum of 9p24.1 alterations in each tumor tissue by FISH assay. At the PD-L1/PD-L2 locus, 9p24.1 amplification was observed in all five patients (Fig. 2).

### Multiplex immunofluorescence

We labeled the TAMs and HRS cells to explore how genetically driven PD-L1 and PD-L2 expression responds to PD-1 axis-targeted therapy in the microenvironmental niche. The anti-CD68 cells were morphologically consistent with TAMs. Malignant HRS cells were identified by a pathologist.

**Fig. 3:**
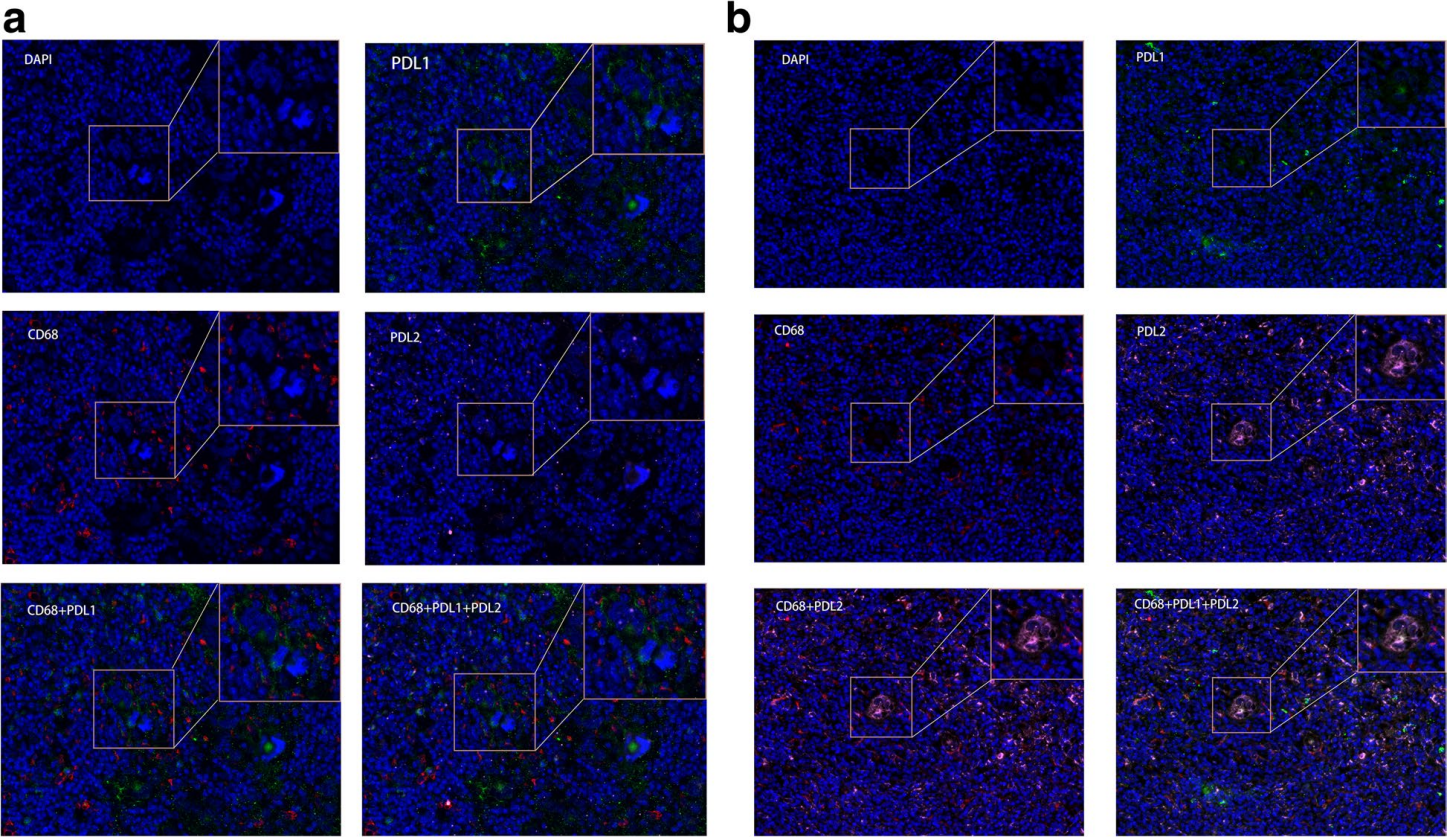
**A** Representative image (40× resolution) from patient 2. PD-L1 (green) and PD-L1 (pink) expression in CD68+ (red) macrophages, HRS cells, and background cells in primary tumors from patient 2. The cells with large nuclei and at least two nuclear lobes are HRS cells. PD-L1 and PD-L2 are colocalized in HRS cells. PD-L1 protein was expressed on the membrane (green) of HRS cells, TAMs, and background cells. PD-L2 was weakly expressed (pink). **B **Representative image (40× resolution) from patient 5. PD-L2 was highly expressed in HRS cells, TAMs, and other background cells in patient 5

Based on the procedure above, we analyzed the expression of PD-L1 and PD-L2 in HRS cells, TAMs, and background cells (Table 2). PD-L1 and PD-L2 were colocalized on HRS cells in all cases except for case 1. In case 2, the expression level of PD-L1 was higher than that of PD-L2 in HRS cells, TAMs, and background cells (Fig. 3 A). The PD-L1+ HRS cells were surrounded by several TAMs and PD-L1+ background cells, and were in close contact. In case 5, PD-L2 was more highly expressed in HRS cells, TAMs and background cells (Fig. 3B). PD-L2+ TAMs contacted the PD-L2+ HRS cells indirectly through their interactions with PD-L2+ background cells. The other three cases all demonstrated differential expression of the biomarkers in background cells. The results showed differential expression of PD-L1 and PD-L2 in cells in cHL, especially in HRS and background cells, as well as TAMs; all of these cells are in contact with each other (Supplementary Fig. 2).

**Table.2:** Classification of cells in the microenvironmental niche based on immunofluorescence staining

Patient	CD68+ macrophages			HRS cells					Background cells						
	CD68	PD-L1	PD-L2	PD-L1	PD-L2	PD-1	MHC-I	MHC-II	PD-L1	PD-L2	PD-1	MHC-I	MHC-II	FOXP3	CD163
1	+	-	-	-	-	-	-	-	++	+	+	++++	Weak+	+	Weak+
2	++	+	-	+	Weak+	-	-	-	+	Weak+	+	+++	+	+++	Weak+
3	+	Weak+	-	+	Weak+	-	-	-	+	Weak+	Weak+	++++	-	+	Weak+
4	+	-	-	+	++	+	+++	-	+	+++	+	++++	+	+	Weak+
5	+	-	+	Weak+	++	-	-	-	Weak+	++	+++	++++	Weak+	++	+

In addition, our results revealed that PD-1 was expressed in background cells in all cases (100%). High MHC class I expression was seen in all cell types, while MHC class II expression was mostly decreased/absent expressed (80%, 4/5) on the background cells. MHC class I+ HRS cells were present only in one of the five patients (20%). MHC class II expression was negative in HRS cells (Table 2).

## Discussion

Targeted immune checkpoint molecular drugs have demonstrated satisfactory efficacy for R/R cHL. However, in clinical practice, PD-L1 and PD-1 inhibitors showed significant differences in treatment efficacy, with the latter eliciting better responses [7–9]. We retrospectively reviewed five patients with R/R cHL treated with a PD-1 inhibitor after disease progression during treatment with a PD-L1 inhibitor. PD-L1 and PD-L2 were colocalized in HRS cells in patients with cHL. Differential expression of PD-L1 and PD-L2 was observed in cells in the TME, especially HRS and background cells.

In R/R cHL, PD-1 inhibitor blockade of interactions with PD-L1 and PD-L2 receptors had an ORR of 66–87%^8,9^, while PD-L1 inhibitors blocking only the PD-L1/PD-1 immune checkpoint had limited efficacy in the iMATRIX trial (atezolizumab) [7] and a phase II clinical trial (CS1001; clicnialtrials.gov identifier :NCT03505996). Some PD-L1-negative patients with other malignancies occasionally show a good clinical response to PD-1 checkpoint blockade [4]. Based on the research above and our results, we believe that blocking the PD-1 pathway completely may lead to better treatment outcomes in cHL than blocking either the PD-L1 or PD-L2 pathway.

PD-L2 was detected in HRS cells and monocytes/macrophages in the TME [14]. PD-L2 is expressed at a lower rate than PD-L1 in HRS cells (41% vs. 82%) [15] due to genetic factors [16]. In HRS cells, 9p24.1 disomy, polysomy, copy gain, amplification and chromosomal rearrangement were noted in this study. Previously, a highly significant negative association was found between residual 9p24.1 disomy and PD-L2 expression [2]. Yearley et al. [17] reported a greater response to pembrolizumab in patients positive for both PD-L1 and PD-L2 (27.5%) compared to those who were positive only for PD-L1 (11.4%), among 172 patients with neck squamous cell carcinoma. PD-L2 status was also a strong predictor of PFS, independent of PD-L1 status, in patients treated with pembrolizumab. Longer median PFS and overall survival (OS) were observed in PD-L2-positive than PD-L2-negative patients. Tanegashima et al. [18] conducted a study on the immunosuppressive role of PD-L2 in PD-1 signal blockade therapy, in both animal models and humans. In animal models, antitumor immune responses were significantly suppressed by PD-L2 expression, alone or coexpressed with PD-L1 in tumor cells. PD-L2 expression was also involved in resistance to treatment with anti-PD-L1 mAb alone, which was overcome by anti-PD-1 mAb, alone or combined with anti-PD-L2 mAb. Antitumor immune responses were significantly correlated with PD-L2 expression in the TME in renal cell carcinoma and lung squamous cell carcinoma.

We found that PD-L2 was expressed in HRS cells in four out of five (80%) cases, and was preferentially expressed compared to PD-L1 in two cases. Background cells were all PD-L2-positve. These results may explain why anti-PD-L1 monotherapy failed in these patients. Due to the differential expression of PD-L1 and PD-L2 in cells in the TME, especially in HRS and background cells, blocking only PD-1 and PD-L1 interactions with PD-L1 monotherapy may disrupt PD-1 and PD-L2 interactions, further weakening the anti-tumor effect. In support of this hypothesis, all of our patients exhibited a dramatic response after blockade of the PD-1 pathway with anti-PD-1 monotherapy.

According to the literature, MHC class I and 2 expression in HRS cells was decreased or abolished in 79% and 67% of cHL patients, respectively. Patients showing decreased or abolished beta 2 M/MHC class I expression in HRS cells had a shorter PFS [19]. MHC class II positivity in HRS cells may predict a favorable outcome of PD-1 blockade [5]. In our study, MHC class I+ HRS cells were present in only one of the five patients (20%), while MHC class II expression was absent in HRS cells in all cases, largely in accordance with the reports above. In addition, high MHC class I expression was seen in background cells, while MHC class II expression was decreased/absent expression (80%, 4/5). All four patients responded well to PD-1 inhibitor. Hence, we infer that MHC class I–II-mediated antigen presentation in the TME (other than in HRS cells) also plays an important role in the treatment response.

Tissue samples from cHL patients show small numbers of atypical germinal center-derived B-cells (HRS cells) in an inactive inflammatory/immune milieu. The TME may determine the anti-tumor response in cHL to a greater degree than PD-L1 expression in tumor cells. As a major component of tumor immune cells, macrophages can be classified into tumoricidal M1-like macrophages and pro-tumoral M2-like macrophages [20]. Macrophages appear to play a major role in tumor growth [21]. Klein et al. reported that, at a cutoff of “25% mean macrophage reactivity”, a statistically significant difference in OS was seen for CD163 (P = .0006) but not for CD68 (P = .414) [10]. Another study showed less intense CD163 than CD68 staining, and weak non-specific staining of background inflammatory and Hodgkin cells [22]. FOXP3 expression may reflect direct suppression of malignant B cells by Tregs in cHL, or the suppression of tumor-supporting T cells in the microenvironment. Clinically, FOXP3 cell density was useful to discriminate among prognostic groups; the group with the most favorable prognosis had the highest FOXP3+ density [11]. In our study, staining was less intense for CD163 than CD68, with relatively weak non-specific staining of background cells. FOXP3 expression was variable. The roles of CD163 and FOXP3 in the TME of cHL require more investigation.

Limitations of the present study included its retrospective design and limited number of cases. In addition, all tissue specimens were obtained at diagnosis, i.e., there were no specimens from the relapse and refractory periods.

## Conclusions

PD-L1 and PD-L2 were colocalized in HRS cells in most of our patients, and simultaneously expressed in background cells. PD-L1 and PD-L2 showed differential expression, especially in HRS and background cells. The immunologic function of the PD-L2 pathway in anti-tumor activity may be underestimated in R/R cHL. Further study is needed to elucidate the anti-tumor mechanism of PD-1 and PD-L1 inhibitor treatment in cHL.

## Supplementary Information

**Additional file 1.**

## Data Availability

The datasets of the study are available on request to the correspondent authors.

## Abbreviations

PD-1: programmed cell death 1
PD-L1: programmed cell death ligand 1
PD-L2: programmed cell death ligand 2
cHL: classical Hodgkin lymphoma
R/R: relapsed or refractory
MHC: major histocompatibility complex
HRS cells: Hodgkin Reed-Sternberg
TME: tumor microenvironment
ASCT: autologous stem cell transplantation
TAMs: tumor-associated macrophages
CR: complete response
PR: partial response
ORRs: overall response rates
PFS: progression-free survival
OS: overall survival

## References

[1] Canellos GP ND, Johnson JL (2009). Long-Term Follow-Up Hodgkin’s Lymphoma. N Engl J Med.

[2] Roemer MG, Advani RH, Ligon AH (2016). PD-L1 and PD-L2 Genetic Alterations Define Classical Hodgkin Lymphoma and Predict Outcome. J Clin Oncol.

[3] Carey CD, Gusenleitner D, Lipschitz M (2017). Topological analysis reveals a PD-L1-associated microenvironmental niche for Reed-Sternberg cells in Hodgkin lymphoma. Blood.

[4] Shen X, Zhao B (2018). Efficacy of PD-1 or PD-L1 inhibitors and PD-L1 expression status in cancer: meta-analysis. BMJ.

[5] Roemer MGM, Redd RA, Cader FZ (2018). Major Histocompatibility Complex Class II and Programmed Death Ligand 1 Expression Predict Outcome After Programmed Death 1 Blockade in Classic Hodgkin Lymphoma. J Clin Oncol.

[6] Hollander P, Kamper P, Smedby KE (2017). High proportions of PD-1(+) and PD-L1(+) leukocytes in classical Hodgkin lymphoma microenvironment are associated with inferior outcome. Blood Adv.

[7] Geoerger B, Zwaan CM, Marshall LV (2020). Atezolizumab for children and young adults with previously treated solid tumours, non-Hodgkin lymphoma, and Hodgkin lymphoma (iMATRIX): a multicentre phase 1-2 study. Lancet Oncol.

[8] Ansell SM, Lesokhin AM, Borrello I (2015). PD-1 blockade with nivolumab in relapsed or refractory Hodgkin’s lymphoma. N Engl J Med.

[9] Armand P, Engert A, Younes A (2018). Nivolumab for Relapsed/Refractory Classic Hodgkin Lymphoma After Failure of Autologous Hematopoietic Cell Transplantation: Extended Follow-Up of the Multicohort Single-Arm Phase II CheckMate 205 Trial. J Clin Oncol.

[10] Klein JL, Nguyen TT, Bien-Willner GA (2014). CD163 immunohistochemistry is superior to CD68 in predicting outcome in classical Hodgkin lymphoma. Am J Clin Pathol.

[11] Greaves P, Clear A, Coutinho R (2013). Expression of FOXP3, CD68, and CD20 at diagnosis in the microenvironment of classical Hodgkin lymphoma is predictive of outcome. J Clin Oncol.

[12] Oike N, Kawashima H, Ogose A (2018). Prognostic impact of the tumor immune microenvironment in synovial sarcoma. Cancer Sci.

[13] Schreck S, Friebel D, Buettner M (2009). Prognostic impact of tumour-infiltrating Th2 and regulatory T cells in classical Hodgkin lymphoma. Hematol Oncol.

[14] Vari F, Arpon D, Keane C (2018). Immune evasion via PD-1/PD-L1 on NK cells and monocyte/macrophages is more prominent in Hodgkin lymphoma than DLBCL. Blood.

[15] Panjwani PK, Charu V, DeLisser M (2018). Programmed death-1 ligands PD-L1 and PD-L2 show distinctive and restricted patterns of expression in lymphoma subtypes. Hum Pathol.

[16] Green MR, Monti S, Rodig SJ (2010). Integrative analysis reveals selective 9p24.1 amplification, increased PD-1 ligand expression, and further induction via JAK2 in nodular sclerosing Hodgkin lymphoma and primary mediastinal large B-cell lymphoma. Blood.

[17] Yearley JH, Gibson C, Yu N (2017). PD-L2 Expression in Human Tumors: Relevance to Anti-PD-1 Therapy in Cancer. Clin Cancer Res.

[18] Tanegashima T, Togashi Y, Azuma K (2019). Immune Suppression by PD-L2 against Spontaneous and Treatment-Related Antitumor Immunity. Clin Cancer Res.

[19] Roemer MG, Advani RH, Redd RA (2016). Classical Hodgkin Lymphoma with Reduced beta2M/MHC Class I Expression Is Associated with Inferior Outcome Independent of 9p24.1 Status. Cancer Immunol Res.

[20] Mantovani A, Sozzani S, Locati M (2002). Macrophage polarization: tumor-associated macrophages as a paradigm for polarized M2 mononuclear phagocytes. Trends Immunol.

[21] Hagemann T, Lawrence T (2009). Investigating macrophage and malignant cell interactions in vitro. Methods Mol Biol.

[22] Harris JA, Jain S, Ren Q (2012). CD163 versus CD68 in tumor associated macrophages of classical Hodgkin lymphoma. Diagn Pathol.

