# Supplementary material for: High efficacy of PD-1 inhibitor after initial failure of PD-L1 inhibitor in Relapsed/Refractory classical Hodgkin Lymphoma
Source: BMC Cancer. 2022 Jan 3;22:9. doi: 10.1186/s12885-021-09028-4 (PMC8722342; doi:10.1186/s12885-021-09028-4)
Supplement: Supplementary file 1 — Additional file 1. [file 12885_2021_9028_MOESM1_ESM.docx]

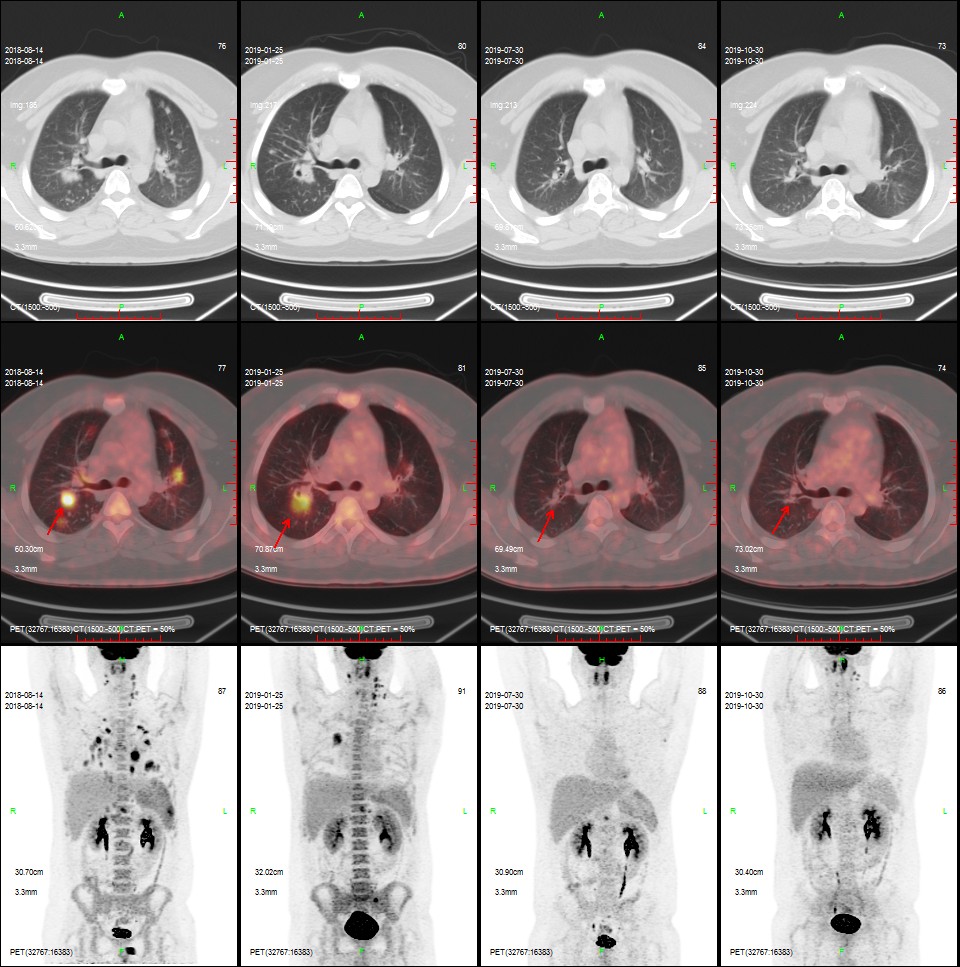


A B C D

Supplementary Figure 1A PET-CT images of patient 1 (stage IV cHL). Images in the top, middle and bottom rows are CT, fusion, and PET images, respectively. A: Panel A shows images taken before anti-PD-L1 therapy, indicating elevated FDG uptake of Waldeyer's ring, lymph nodes of the bilateral neck, the left supraclavicular region, mediastinum, and bilateral hilar region, and the hepatic portal, extraperitoneal space, bilateral lungs, and spleen. B: Panel B shows PET-CT images taken after four cycles of anti-PD-L1 therapy. Compared to that of pre-PD-L1, the FDG uptake of most lymph nodes with lymphoma increased, with a Deauville score of 4–5. Most lesions in the bilateral lungs shrunk, but a nodule located in the right lung was enlarged (red arrow). C: Panel C shows images taken after four cycles of PD-1 inhibitor. In general, PET-CT revealed significant remission. The only remaining lymph node in the extraperitoneal space had also shrunk, showing reduced FDG uptake (Deauville score of 5). D: Panel D images were reviewed during maintenance of anti-PD-1 therapy after autologous stem cell transplantation. Complete remission was achieved.


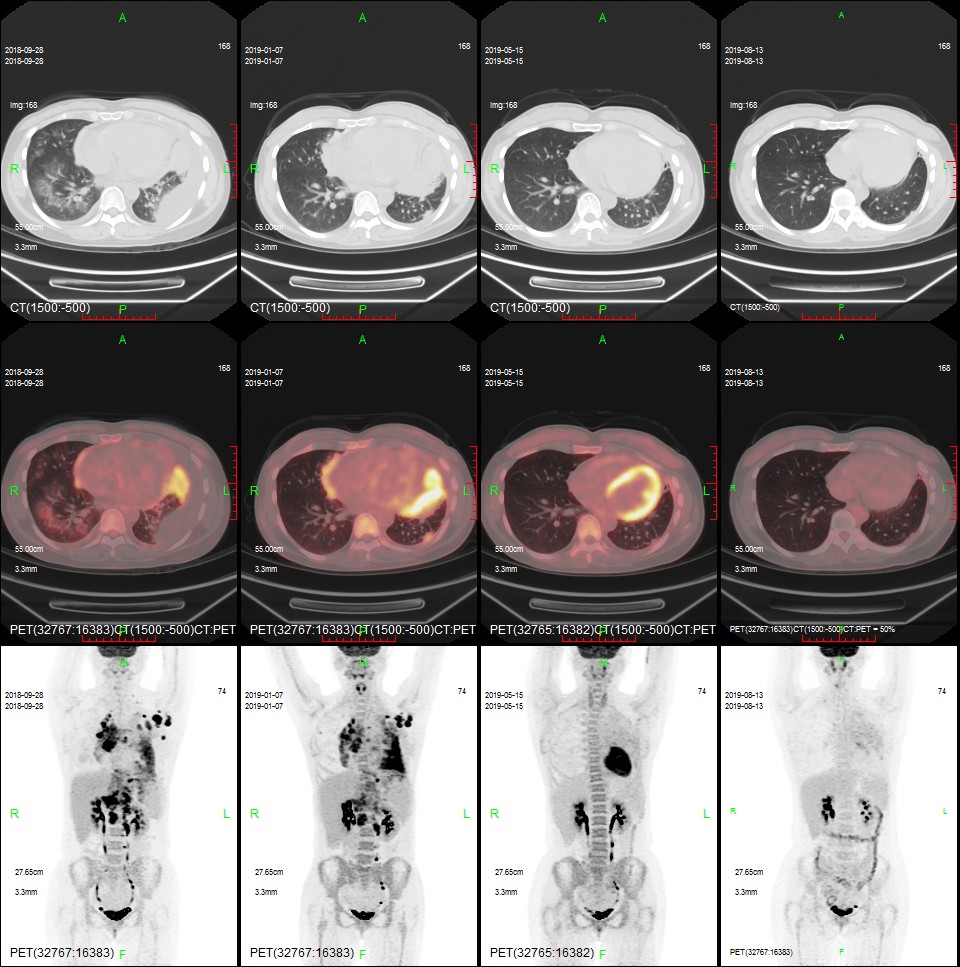


A B C D

Supplementary Figure 1B PET-CT images of patient 2 (stage IV cHL). Images in the top, middle and bottom rows are CT, fusion, and PET images, respectively. A: Panel A images were taken before anti-PD-L1 therapy. The lymph nodes of the left neck, left chest wall, left intermammary area, left axilla, mediastinum, right hilar, hepatic portal, hepatogastric space, extraperitoneal space, left lung, left pleura, pericardium, and liver were involved. B: After anti-PD-L1 treatment, the PET-CT scan indicated increased FDG uptake in the left lung, pleura, pericardium, and liver (Deauville score of 5), which was considered to reflect disease progression. C: Panel C shows images taken after anti-PD-1 therapy. PET-CT revealed significant remission of most lesions, except for the nodule in the left pleura (red arrow), which showed slightly elevated FDG uptake (Deauville score of 4). D: Repeated PET-CT scans during maintenance anti-PD-1 therapy revealed complete and persistent remission.


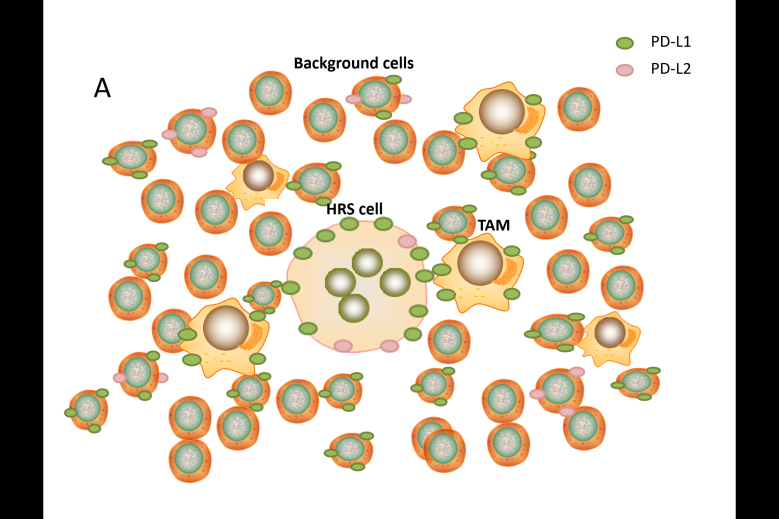

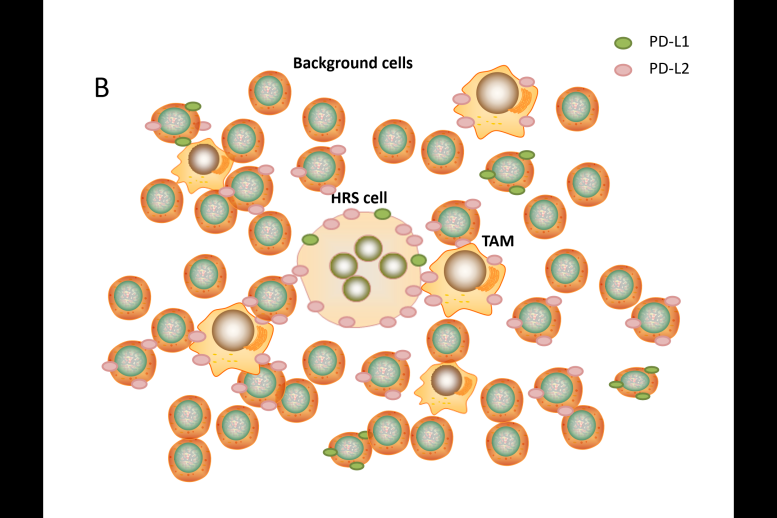


Supplementary Figure 2 Schema of the tumor microenvironment in cHL.
